# Single‐Step Selective Laser Writing of Flexible Photodetectors for Wearable Optoelectronics

**DOI:** 10.1002/advs.201800496

**Published:** 2018-06-06

**Authors:** Jianing An, Truong‐Son Dinh Le, Chin Huat Joel Lim, Van Thai Tran, Zhaoyao Zhan, Yi Gao, Lianxi Zheng, Gengzhi Sun, Young‐Jin Kim

**Affiliations:** ^1^ School of Mechanical and Aerospace Engineering Nanyang Technological University 50 Nanyang Avenue 639798 Singapore; ^2^ Singapore Centre for 3D Printing School of Mechanical and Aerospace Engineering Nanyang Technological University 50 Nanyang Avenue 639798 Singapore; ^3^ Department of Mechanical Engineering Khalifa University of Science, Technology and Research Abu Dhabi 127788 United Arab Emirates; ^4^ Key Laboratory of Flexible Electronics (KLOFE) & Institute of Advanced Materials (IAM) Jiangsu National Synergetic Innovation Center for Advanced Materials (SICAM) Nanjing Tech University (NanjingTech) 30 South Puzhu Road Nanjing 211816 P. R. China

**Keywords:** flexible photodetectors, graphene hybrids, hierarchical morphology, single‐step selective laser writing, wearable optoelectronics

## Abstract

The increasing demand for wearable optoelectronics in biomedicine, prosthetics, and soft robotics calls for innovative and transformative technologies that permit facile fabrication of compact and flexible photodetectors with high performance. Herein, by developing a single‐step selective laser writing strategy that can finely tailor material properties through incident photon density control and lead to the formation of hierarchical hybrid nanocomposites, e.g., reduced graphene oxide (rGO)–zinc oxide (ZnO), a highly flexible and all rGO–ZnO hybrid‐based photodetector is successfully constructed. The device features 3D ultraporous hybrid films with high photoresponsivity as the active detection layer, and hybrid nanoflakes with superior electrical conductivity as interdigitated electrodes. Benefitting from enhanced photocarrier generation because of the ultraporous film morphology, efficient separation of electron–hole pairs at rGO–ZnO heterojunctions, and fast electron transport by highly conductive rGO nanosheets, the photodetector exhibits high, linear, and reproducible responsivities to a wide range of ultraviolet (UV) intensities. Furthermore, the excellent mechanical flexibility and robustness enable the photodetector to be conformally attached to skin, thus intimately monitoring the exposure dosage of human body to UV light for skin disease prevention. This study advances the fabrication of flexible optoelectronic devices with reduced complexity, facilitating the integration of wearable optoelectronics and epidermal systems.

## 1 Introduction

Ultraviolet (UV) photodetectors have demonstrated their broad applications in diverse fields including environmental pollutant analysis, optical communication, space surveillance, and health monitoring.^1^ In particular, wearable and sustainable photodetectors are urgently desired for continuously monitoring the UV irradiation dosage, in order to modulate a person's exposure to the sunlight, thereby promoting the positive effects (e.g., vitamin D synthesis and mental wellness) and preventing the negative effects (e.g., skin aging, skin cancer, eye damage, and immune system suppression) of solar UV radiation on human body.[[qv: 1d,2]] Although conventional UV detection systems based on rigid components are able to display the exposure level, the lack of softness and flexibility has severely restricted their conformal contact with human skin, which makes the devices incompatible with most outdoor activities requiring accurate and accumulative UV dosimetry.[[qv: 1d]] Therefore, novel intelligent photodetectors, which are flexible, deformable, durable, compact, light‐weight, and skin‐like, are of great necessity for the development of wearable healthcare optoelectronics.^3^

To date, flexible photodetectors have been generally manufactured by the sequential deposition of electrodes and active materials on top of polymeric substrates in a layer‐by‐layer manner, which is inadequate for modern wearable optoelectronics.[[qv: 3a,4]] For instance, metal electrodes are traditionally patterned through cumbersome lithography and high‐vacuum deposition processes, which are extremely time‐consuming and suffering from severe contamination issues.[[qv: 1b,5]] Alternatively, conductive inks composed of metal nanoparticles, metal nanowires, and carbon‐based materials have been developed for directly printing the electrodes on flexible substrates.^4^ The drawbacks of these techniques, however, have been revealed in terms of the nozzle clogging by inks with improper feature size, and the high‐temperature post‐sintering steps for improving the electrical interconnection which are incompatible with most polymeric substrates having low melting temperature.^4^, ^6^ In the case of active materials, great efforts have been dedicated to construct 3D hierarchical semiconductors with abundant heterojunctions via hybridization, which are expected to enhance the absorption of incident light and reduce the recombination of photogenerated electron–hole pairs, thus improving the responsivity.^7^ Conventionally, hydrothermal method is adopted to prepare the hybrid nanostructures, however the nanostructure aggregation is inevitable due to the high reaction temperature and elongated reaction time. Furthermore, the post‐synthesis dispersion of the nanocomposites in solution and the subsequent deposition on flexible substrates make it difficult to retain the as‐formed 3D hierarchical structure. These lead to a reduced surface area and consequently limit the performance of the photodetectors.^8^ On the other hand, although chemical vapor deposition has been proven to be an effective approach to obtain heterostructures with high quality, the multistep growth and post‐transfer processes make it complicated and inefficient for scalable fabrication.^9^ In addition, the routine procedure of preparing flexible devices is to separately assemble the electrodes and the active materials on flexible platforms, therefore, both of the interfacial bonding between electrodes and active materials and their affinity to the flexible substrates are weak, resulting in unexpected delamination and detachment of the device structures during cyclic deformation, thus greatly degrading the mechanical robustness and durability of the devices.[[qv: 1b,2b,10]]

Aiming to tackle the aforementioned problems, we herein report a single‐step selective laser writing strategy to fabricate highly flexible UV photodetectors in a designable, maskless, transfer‐free, and eco‐friendly manner. By controlling the incident photon density (scanning speed of the femtosecond laser), hierarchical reduced graphene oxide (rGO)–zinc oxide (ZnO) hybrid nanocomposites with tunable electrical and optoelectrical properties were successfully obtained. It is noteworthy that the femtosecond laser, owing to its ultrashort pulse duration and high peak power, serves as a localized heat source to effectively photoreduce GO and form ZnO nanoparticles (NPs), while avoiding the thermal damage of flexible substrates by minimizing the heat diffusion.^11^ Benefited from above merits, in‐plane all rGO–ZnO hybrid‐based photodetectors were designed directly on flexible substrates, where 1) the interdigitated electrodes composed of relatively densely packed and deeply photoreduced rGO nanoflakes anchored with poor crystalline ZnO NPs are highly conductive, thus guaranteeing the fast carrier transport to external circuit; 2) the active detection layer features the morphology of 3D ultraporous carbon skeleton decorated with high crystalline ZnO NPs, which not only improves the photocarrier generation through the enhanced light absorption and oxygen molecules penetration and adsorption but also accelerates the electron–hole pairs separation due to the formation of rGO–ZnO heterojunctions. As a result, the as‐fabricated flexible and all rGO–ZnO hybrid‐based photodetector exhibited a high, linear, and reproducible UV responsivity over a wide intensity range at a low operation voltage, together with excellent mechanical robustness and durability. This simple and straightforward single‐step fabrication also demonstrates its potentials for the multiscale production and integration of devices with reduced complexity and cost, paving the way for efficient manufacturing of wearable and smart optoelectronics.

## 2 Results and Discussion

**Figure 1:**
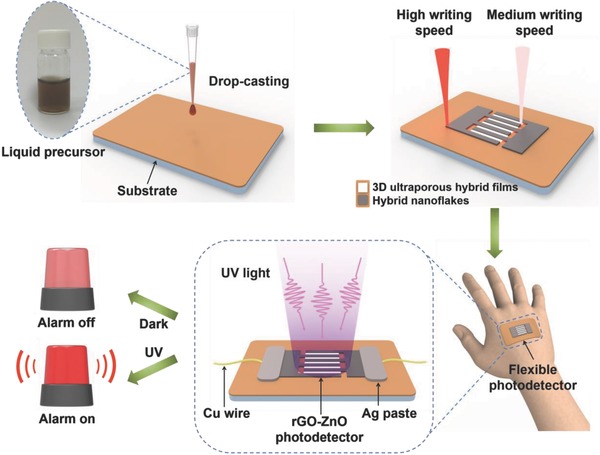
Schematic illustration of the preparation of all rGO–ZnO hybrid‐based photodetector through a single‐step FsLDW process by selecting suitable writing speed for respectively patterning the interdigitated electrodes and the active detection layer. The flexible photodetector can be used for human skin protection.

The overall single‐step fabrication of the all rGO–ZnO hybrid photodetectors is schematically outlined in **Figure** **1**. Briefly, GO nanosheets aqueous suspension and zinc acetate (Zn(CH_3_COO)_2_∙2H_2_O) solution were mixed by a simple solution blending method at the volume ratio of 1:4. The homogeneous precursor mixture was achieved by gentle magnetic stirring. After coating a uniform film of the precursor onto a flexible substrate, femtosecond laser direct writing (FsLDW) was performed on the film, enabling the photoreduction of GO to rGO and thermal decomposition of Zn(CH_3_COO)_2_∙2H_2_O to ZnO simultaneously (see Chemical Reactions S1–S3 in the Supporting Information). Since FsLDW is capable of finely modulating the properties of the as‐synthesized materials through precisely tuning the photon/phonon energy absorbed by the precursors, the all rGO–ZnO hybrid photodetectors can be fabricated in a single step by selecting suitable writing speed for patterning the active detection layer and the electrodes, respectively. Due to its high flexibility and mechanical robustness, thus‐fabricated photodetector can be conformally attached to human body and would be a promising candidate as smart alerting system to protect human body from overdosing of UV exposure, as illustrated in Figure 1.

**Figure 2:**
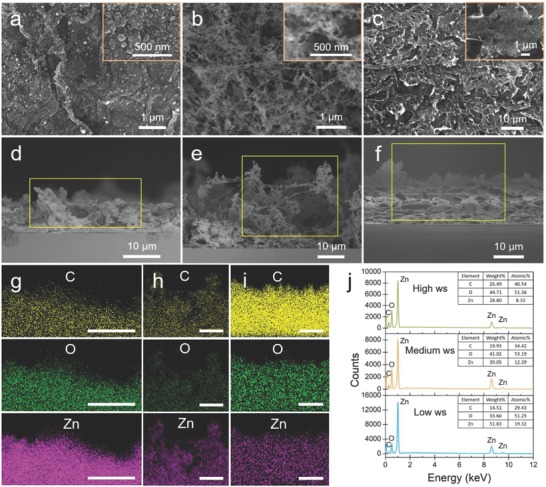
Morphology and chemical composition modulation of the rGO–ZnO heterostructures by the writing speed. a–c) SEM images (top surface) of the hybrids produced under low, medium, and high writing speeds, respectively. The insets are top views at a higher magnification. d–f) Cross‐section SEM images of the nanostructures synthesized under low, medium, and high writing speeds, respectively. Their corresponding EDS element (C, O, and Zn) mapping profiles are respectively displayed in panels (g)–(i), scale bar: 10 µm. j) EDS spectra of the three rGO–ZnO hybrids, the insets present the element analysis in weight and atomic percentage.

In this study, three levels of writing speeds were utilized to controllably prepare rGO–ZnO hybrid under identical laser power of 120 mW. The levels are categorized here as low writing speed (typically at 1 mm s^−1^), medium writing speed (typically at 10 mm s^−1^), and high writing speed (typically at 100 mm s^−1^). The scanning electron microscopy (SEM) images in **Figure** **2** reveal that rGO–ZnO heterostructures with different hierarchical morphologies were produced when varying the writing speed. Under low writing speed (Figure 2a,d), wrinkled rGO films densely packed with quasispherical ZnO NPs were formed. When the writing speed was increased to 10 mm s^−1^, ultraporous carbon skeleton decorated with ZnO NPs was synthesized (Figure 2b,e). Under high writing speed (Figure 2c,f), carbon networks that are composed of rGO nanoflakes anchored with small amount of ZnO NPs were produced. The energy dispersive X‐ray spectroscopy (EDS) element mapping profiles (Figure 2g–i) clearly confirm that each constituent atom (C, O, and Zn) is uniformly distributed throughout the heterostructures from the surface to the interior for all rGO–ZnO nanocomposites. It is noteworthy that with the decrease of the writing speed, the weight percent and atomic percent of C drop while those of Zn rise (Figure 2j), indicating strong ablation of carbon at lower writing speed. When the exposure time was further elongated by repetitively laser writing the film at the speed of 0.1 mm s^−1^ for 20 times, ZnO NPs were aggregated into clusters, and the weight percent of C is only 3.03% in the hybrid (Figure S1 in the Supporting Information), providing further evidence on carbon ablation under extreme condition. In addition, the thickness variation (Figure S2 in the Supporting Information) of the rGO–ZnO hybrid films also indicates that the laser‐induced thermal ablation dominates in the low writing speed regime (the thickness of the as‐written film decreases compared to that of the precursor film), while the rapid laser scanning promotes the thermal expansion, resulting in the increase in film thickness due to the liberation of interior gaseous products.^11^, ^12^

**Figure 3:**
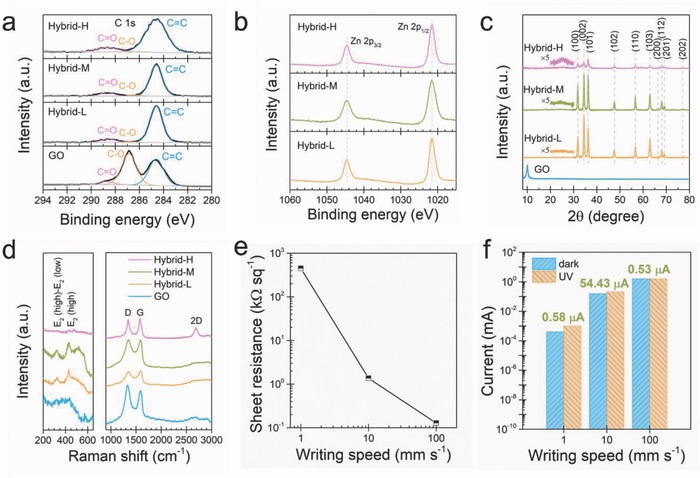
Characterizations of the rGO–ZnO hybrids. a) XPS C1s spectra of GO and rGO–ZnO hybrids. b) XPS Zn2p spectra of rGO–ZnO hybrids. c) XRD patterns of GO and rGO–ZnO hybrids. d) Raman spectra of GO and rGO–ZnO hybrids. e) Sheet resistances of the rGO–ZnO films produced at three levels of writing speed. f) Photoresponse behaviors of the three rGO–ZnO hybrids. The hybrid‐M generates the largest photocurrent.

For simplicity, the hybrids produced under low (1 mm s^−1^), medium (10 mm s^−1^), and high (100 mm s^−1^) writing speeds are respectively denoted hereafter as hybrid‐L, hybrid‐M, and hybrid‐H. X‐ray photoelectron spectroscopy (XPS) was utilized to characterize the photoreduction of GO and the formation of ZnO in the hybrids. The high‐resolution XPS C1s spectrum of GO can be deconvolved into three peaks (**Figure** **3**a), centered at 284.6 (C=C group), 286.9 (C—O group), and 288.7 eV (C=O group), respectively. For comparison, the deconvoluted species of all rGO–ZnO hybrids are respectively located at 284.6 (C=C group), 286.8 (C—O group), and 288.6 eV (C=O group). The intensities of all the oxygen‐related peaks reduce dramatically, indicating the successful removal of oxygen‐containing groups from GO and the deep photoreduction of GO by FsLDW.^11^, ^13^ In addition, the high‐resolution Zn2p spectra of rGO–ZnO hybrids (Figure 3b) show two prominent peaks at around 1021.47 (Zn2p_1/2_) and 1044.56 eV (Zn2p_3/2_), respectively, implicating the formation of ZnO.[[qv: 8a,14]] The crystal structure of the pristine GO and the hybrids written under three conditions were examined using X‐ray diffraction (XRD), and the patterns are displayed in Figure 3c. The (002) diffraction peak of GO film is located at 10.1°, indicating the interplane distance of around 0.88 nm. For the rGO–ZnO hybrids, the intense GO peak disappears, while a broad peak appears at 24° and becomes more prominent for the hybrid produced at higher writing speeds. All the other detectable peaks of the rGO–ZnO hybrids can be indexed to the hexagonal wurtzite ZnO (JPCDS card No. 36‐1451) structure.^15^ The ZnO peaks of the hybrid‐L and hybrid‐M are intense and sharp, indicating a good crystalline quality of the ZnO NPs. On the contrary, the ZnO peak intensities decrease significantly for the hybrid‐H, implicating the crystallinity of the ZnO NPs is poor, which may be resulted from the insufficient sintering temperature because the exposure time to the laser irradiation is rather short.[[qv: 7a]] As a powerful tool for structural characterization of carbonaceous materials,^16^ Raman spectroscopy was employed to study the characteristics of GO and rGO–ZnO hybrids. The Raman spectrum of GO, as shown in Figure 3d, features two dominant peaks which correspond to the D band at 1332 cm^−1^ and the G band at 1594 cm^−1^, respectively. For all rGO–ZnO hybrids, the D band blueshifts while the G band redshifts, implying the reduction of GO to rGO after laser irradiation.^12^ In addition, the intensity ratio of D band and G band (*I*_D_/*I*_G_) of the hybrids reduces compared to that of GO, clearly indicating the decrease in structural defects and the removal of oxygen‐containing groups by the laser irradiation. Notably, an intense and sharp 2D band, which is associated with the generation of crystalline graphitic materials, is observed at 2683 cm^−1^ for hybrid‐H. This result evidences the effective photoreduction of GO to rGO by the rapid laser scanning, which will be beneficial for a fast electron transfer due to the readily restored electrical conductivity.^12^ Meanwhile, the formation of ZnO NPs in the hybrids by FsLDW was confirmed in the low frequency region of the Raman spectra (Figure 3d). Two prominent peaks are observed from hybrid‐L and hybrid‐M, which are assigned to the E_2_ (high)‐E_2_ (low) mode at 325 cm^−1^ and the E_2_ (high) mode at 431 cm^−1^, respectively. By contrast, the peaks are negligible for hybrid‐H, suggesting the poor crystallinity of the ZnO NPs. This result is consistent with the XRD analyses that a slow scanning will elongate the laser irradiation time on the precursor film, thus raising the local temperature for the formation and crystallization of ZnO NPs.

The electrical and photoresponsive properties of the as‐synthesized rGO–ZnO hybrids were probed subsequently. As displayed in Figure 3e, the sheet resistance, which was measured using a four‐probe method,^11^, ^17^ is as high as 0.45 MΩ per square for hybrid‐L, and decreases to 1.36 kΩ per square for hybrid‐M. Intriguingly, the sheet resistance is only 128 Ω per square for hybrid‐H, which can be attributed to the high level of graphitization of the nanostructures by rapid laser scanning, as proven by the aforementioned materials characterizations. 5 mm × 1 mm rectangles were patterned at three levels of writing speed on three precursor films, for testing the photoresponse of the as‐written films by measuring the current–voltage (*I*–*V*) curves in the dark and under UV illumination (light intensity of 20.03 mW cm^−2^ at 365 nm), respectively. As shown in Figure S3 (Supporting Information), all *I*–*V* curves show linear and symmetric behaviors, indicating that the electrode‐hybrid contacts are Ohmic. For the hybrid‐L (Figure S3a in the Supporting Information), the output currents (at 1 V bias) in the dark (*I*_dark_) and under UV illumination (*I*_UV_) are both very low (*I*_dark_ = 0.39 µA and *I*_UV_ = 0.97 µA), producing a small photocurrent (*I*_ph_ = *I*_UV_ − *I*_dark_) of 0.58 µA, as plotted in Figure 3f. In comparison, hybrid‐M exhibits a much stronger photoresponse to the UV illumination. As shown in Figure S3b (Supporting Information), the current steady state at 1 V is 153.23 µA in the dark and 207.66 µA under UV illumination, thus generating a large *I*_ph_ of 54.43 µA (Figure 3f). By contrast, the *I*–*V* curves of hybrid‐H in the dark and under UV illumination almost overlap each other (Figure S3c in the Supporting Information), where a small *I*_ph_ of 0.53 µA is induced at 1 V bias (Figure 3f). The large differences in electrical conductivity and photoresponse of the three hybrids could be correlated with their nanostructures in which rGO nanosheets and ZnO NPs are expected to facilitate the intra & interlayer electrical connections and the UV light absorption, respectively.^15^ According to preceding structural characterizations, hybrid‐L exhibits a high electrical resistance due to the strong thermal ablation of rGO nanosheets, therefore the photogenerated charges cannot be effectively transported to external circuit, consequently both of the *I*_dark_ and *I*_UV_ are very low. Under high writing speed, although the photoreduction of GO is promoted, the poor crystalline ZnO NPs lead to a negligible *I*_ph_, as a result, *I*_dark_ and *I*_UV_ are almost the same and their high values are due to the high conductivity of rGO.^15^ The medium writing speed produces the nanostructures consisting of 3D ultraporous carbon skeleton and decorated ZnO NPs with good crystallinity. Such a heterostructure provides considerable conductivity and effective UV detection property, thereby generating an enhanced photocurrent compared to the other two hybrids.

**Figure 4:**
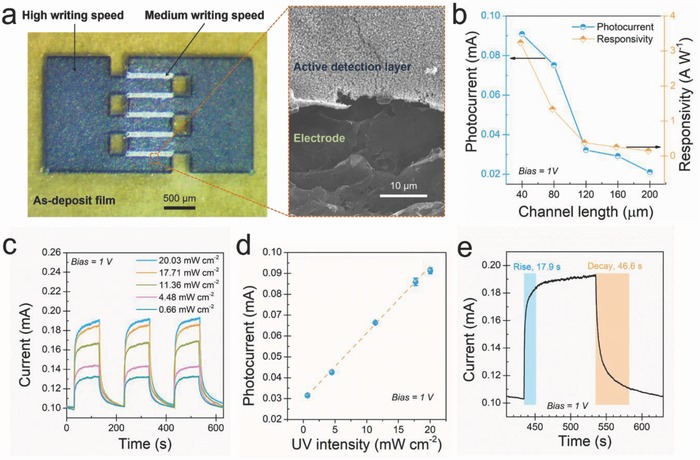
Interdigitated photodetector fabricated by the single‐step FsLDW and the photoresponse performances. a) An optical image of the as‐written interdigitated photodetector, an enlarged SEM image indicates the active detection layer and the electrode. b) The photocurrent and the responsivity as a function of the channel length. c) Time‐resolved photocurrent responses of the photodetector (channel length of 40 µm) to different UV illumination intensities at a bias of 1 V. d) The dependence of photocurrent on incident light intensity. e) Time response of the same device under UV illumination with the light intensity of 20.03 mW cm^−2^.

By leveraging the peculiar characteristics offered by FsLDW, we managed to fabricate the all rGO–ZnO hybrid UV photodetectors in a single‐step selective laser writing process, where the electrodes were written at high speed to obtain high conductivity while the active detection layer was written at medium speed for superior UV detection capability. Interdigitated electrodes with finger length of 1 mm and finger width of 0.3 mm were patterned on the as‐prepared precursor film, as shown in **Figure** **4**a. The channel width was fixed as 0.7 mm, while the channel length (the spacing between two fingers) can be freely tuned by the design. The active detection layer and the electrodes can be clearly distinguished in the SEM image (see Figure 4a). We first studied the effect of channel length on the photocurrent response of the photodetector. As shown in Figure 4b, the device with channel length of 40 µm exhibits high responsivity (*R* = *I*_ph_/*P*, where *P* is the incident UV light power^18^) of 3.24 A W^−1^ toward UV illumination (light intensity of 20.03 mW cm^−2^) at the operation voltage of 1 V. This responsivity is superior to that of densely packed ZnO NP‐based UV photodetectors (0.1 mA W^−1^ at 1 V),^19^ comparable to that of ultraporous ZnO NP network‐based devices (13 A W^−1^ at 5 V and 6.8 A W^−1^ at 1 V),[[qv: 1c,20]] but inferior to that of interlinked ZnO NP network‐based photodetectors (430 A W^−1^ at 5 V).^21^ As the channel length was elongated to 200 µm (with increment of 40 µm), *I*_ph_ decreases from 91 to 21 µA, and the corresponding responsivity gradually drops to 0.15 A W^−1^. This can be well interpreted due to the accumulated defects in rGO–ZnO hybrid, which leads to enhanced scattering of charge carriers.^22^ To demonstrate the UV photoresponse characteristics of our all rGO–ZnO hybrid photodetector in more details, we extensively investigated the device with channel length of 40 µm under UV illumination with varied intensities. The time‐resolved photocurrent responses (at 1 V bias) with multiple UV on/off cycles are displayed in Figure 4c, the device exhibits consistent and repeatable output currents for a wide range of UV illumination (from 0.66 to 20.03 mW cm^−2^). In addition, the generated photocurrent increases almost linearly with rising UV intensity, as plotted in Figure 4d. By defining the rise time (decay time) as the time required for 90% change of the photocurrent from its minimum (maximum) value, we extracted the rise and decay times to be 17.9 and 46.6 s, respectively, from a single on/off cycle of the current–time curve (as shown in Figure 4e). The response‐recovery performance of the all rGO–ZnO hybrid‐based photodetector is comparable to and even superior to that of most precedent ZnO NP‐based, ZnO nanowire‐based rGO–ZnO nanocomposite‐based, and graphdyine‐ZnO nanocomposite‐based UV photodetectors with metal electrodes deposited through cumbersome lithography processes.[[qv: 1c,7a,23]] However, the rise/decay times are inferior to those of ZnO nanotetrapod‐based devices which benefited from the reduced potential barrier at the boundary of two interpenetrating nanowires.[[qv: 23c]]

**Figure 5:**
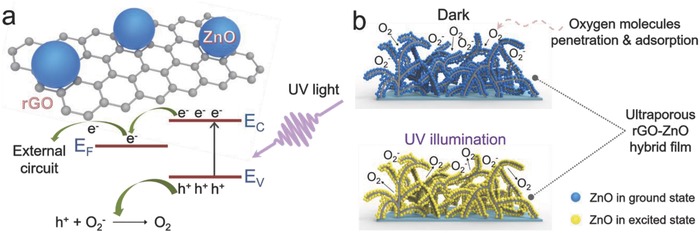
Photoresponse mechanism of the ultraporous all rGO–ZnO photodetector. a) Energy band diagram of the rGO–ZnO hybrid. *E*_C_ and *E*_V_ represent the conduction band and valence band of ZnO, respectively; *E*_F_ represents the Fermi level of rGO. b) Schematic model of the photodetection mechanism of the 3D ultraporous rGO–ZnO hybrid film.

The excellent photoresponse performances of the all rGO–ZnO hybrid‐based photodetector can be well ascribed to the unique material characteristics and the subtle device structure, we thus elaborate the photodetection mechanism as follows. In the active detection layer, high crystalline ZnO NPs are responsible for the chemisorption/photodesorption of oxygen molecules on the ZnO surface and the photogeneration/recombination of electron–hole pairs.^18^, ^24^ In the dark, the ambient oxygen molecules are adsorbed on the ZnO surfaces and ionized to O_2_^−^ by capturing the free electrons from the semiconductor conduction band, thus creating an electron‐depleted layer near the ZnO surface.[[qv: 1c,15,24]] Upon UV illumination, electron–hole pairs are generated in ZnO, as schematically depicted in **Figure** **5**a. The photogenerated holes will migrate to the ZnO surface neutralizing the adsorbed O_2_^−^, thus increasing the free carrier concentration due to the reduction in the depletion layer thickness. The output current of the device is thus dramatically increased upon the UV illumination.[[qv: 1b,c]] The 3D ultraporous structure composing the active detection layer not only aids in the penetration of oxygen molecules into the interior layers and provides increased available surface area for the adsorption of the oxygen molecules (Figure 5b)[[qv: 1c]] but also enables local confinement of the incident UV light inside the film. Therefore, compared to the case of the dense 2D film where most of the light is directly reflected at the top surface, this unique 3D ultraporous architecture ensures the whole film to effectively participate in the photodetection, significantly enhancing the photoresponse.[[qv: 1c]] In the meantime, the electron–hole pairs separation is facilitated with the assist of heterojunctions formed at the interfaces between rGO and ZnO (see Figure S4 in the Supporting Information) by lowering down the recombination rate.[[qv: 22b,25]] After electron–hole pairs separation at the rGO–ZnO heterojunctions, the remaining electrons flow to the Fermi level of rGO through the nanocomposite interfaces.[[qv: 22b,25]] As the intimate electron pathway between the ZnO active material and external circuit, the highly conductive rGO throughout the active detection layer and the electrodes guarantees efficient electron transport, thereby accelerating the photoresponse of the device.^26^

**Figure 6:**
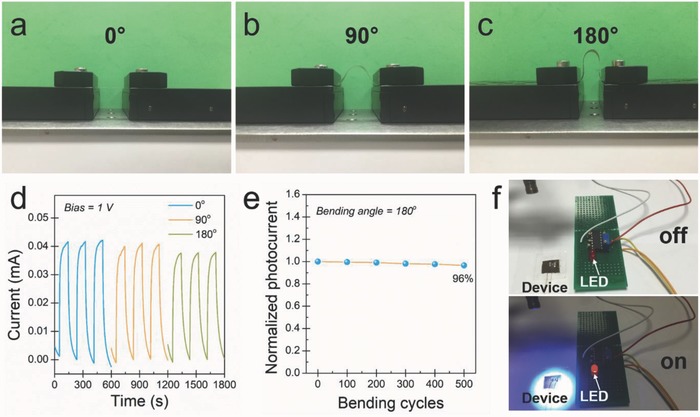
Flexible photodetector and its application as a photoswitch. a–c) Optical images of a flexible photodetector with the bending angle of 0°, 90°, and 180°, respectively. d) Time‐resolved photoresponse curves of the device at different bending angles. e) Normalized photocurrent of the flexible photodetector with respect to the bending–unbending cycles at 1 V bias, the bending angle is 180°. f) Optical images of the photoswitch triggering a red LED upon UV illumination. Upper panel: before UV illumination (LED in the “off” state); lower panel: after UV illumination (LED in the “on” state).

The flexible all rGO–ZnO hybrid‐based photodetector was patterned through a single‐step FsLDW on a bendable mica substrate. Its mechanical flexibility and stability were examined with respect to the bending angles (0°, 90°, and 180°; the corresponding bending radius are ∞, 5.7, and 4.1 mm), as shown in **Figure** **6**a–c. The device exhibits stable cyclic photoresponses even when it was bent to 180° (see Figure 6d), the minor current drop may be induced by the smaller active area under deflected condition.^27^ The excellent flexibility allows for the device to intimately contact with the human hand (see Figure S5 in the Supporting Information), functioning as a wearable photodetector for health monitoring. In addition, as plotted in Figure 6e, the flexible photodetector shows high photocurrent retention (over 96%) after 500 bending–unbending cycles (with the bending angle of 180°), indicating its excellent mechanical stability and reliability. As a simple proof‐of‐concept demonstration of real‐time monitoring the UV radiation in the environment, the flexible photodetector was integrated with an external amplifying circuit for constructing a smart photoswitch system, as described in the Supporting Information (Figure S6, Supporting Information). The resistance variation of the photodetector caused by the UV illumination leads to the corresponding change in the output circuit, which brightens the indicative light‐emitting diode (LED), as shown in Figure 6f and Video S1 (Supporting Information).

## 3 Conclusions

In summary, highly flexible all rGO–ZnO hybrid‐based photodetectors were fabricated through a single‐step selective laser writing strategy by facilely tuning the incident photon energy at different regions of the device. The interdigitated electrodes were prepared by rapid laser scanning which generated highly conductive rGO nanoflakes anchored with poor crystalline ZnO NPs, while the active detection layer was obtained under medium writing speed which formed the ultraporous carbon skeleton decorated with high crystalline ZnO NPs. The as‐fabricated photodetector exhibited high, linear, and reproducible UV responsivities over a wide intensity range at a low operation voltage. The superior performances can be attributed to two factors: 1) the efficient photocarrier generation contributed by the 3D ultraporous films which enhance the absorption of incident light and the penetration and adsorption of oxygen molecules into the films; and 2) the facile separation of electron–hole pairs by the rGO–ZnO heterojunctions and the fast electron transport by the rGO nanosheets. The flexible photodetector also showed excellent mechanical robustness, which is critical for wearable optoelectronic devices. A smart flexible photoswitch prototype was demonstrated to prove its potential applications in real‐time environmental monitoring of UV hazards, holding great promises for various applications in public safety, personal protection, and healthcare.

## 4 Experimental Section

*Preparation of Precursor Films*: GO nanosheets were produced from natural graphite powder (Fluka 50870) via a modified Hummers' method and dispersed in deionized (DI) water with a concentration of 1 mg mL^−1^ using bath sonication, as shown in the atomic force microscopy (AFM) image (Figure S7 in the Supporting Information). 0.1 m of zinc acetate solution was prepared by dissolving 219.5 mg of Zn(CH_3_COO)_2_ ∙ 2H_2_O (Sigma‐Aldrich) into 10 mL of DI water. Then, the GO dispersion and the metal salt solution were mixed with a solution volume ratio of 1:4 under gentle stirring to achieve a homogeneous precursor mixture. After degassing for 1 h, the liquid precursor was drop‐casted on well‐cleaned substrates with a pipette and dried on a hotplate at 40 °C for 2 h, forming a uniform hybrid film.

*Fabrication of All rGO–ZnO Hybrid‐Based Photodetectors*: A programmable FsLDW platform was employed for the fabrication of photodetectors, the setup was described in our previous work.^11^ Briefly, as shown in Figure S8 (Supporting Information), the near‐infrared femtosecond laser beam (center wavelength: 780 nm, pulse width: 100 fs, repetition rate: 83 MHz; Toptica, Femtofiber Pro NIR) was directed onto a 2D plane through an *f*‐theta lens. The focus spot size was estimated to be around 40 µm. The laser beam was steered by a two galvano‐mirror system, and raster‐scanned on the sample surfaces. The laser power was fixed at 120 mW for all scans, the writing speed can be facilely tuned up to 1 m s^−1^.

*Characterizations*: The morphologies of the hybrids were examined by a field‐emission scanning electron microscopy (FESEM, Jeol JSM‐7600F), the EDS element mapping and spectra were acquired by the equipped EDS spectrometer (Jeol JED‐2300F) with an acceleration voltage of 15 kV. XRD patterns were recorded using a PANalytical Empyrean diffractometer with a Cu Kα (λ = 1.54 Å) irradiation source. Raman spectra were collected in the range of 150–3000 cm^−1^ using a Renishaw InVia confocal Raman microscope with a 514 nm laser excitation line at a power of 0.5 mW. XPS was carried out on a PHI‐5400 spectrometer with a monochromatic Al Kα source (*hν* = 1486.6 eV). The binding energies were corrected to C=C species at 284.6 eV as reference. The film thickness measurements were conducted on a 3D laser scanning microscope (Keyence VK‐X250). The sheet resistances of the as‐prepared films were evaluated using a four‐probe method based on the van der Pauw model.^17^

*Device Measurements*: The *I–V* curves were measured in the dark and under UV illumination using a Keysight B2902A source/measure unit (SMU). The time‐resolved responses of the photodetectors to the UV “on” and “off” were recorded on the SMU with 100 ms per point integration time. A UV LED with the center wavelength at 365 nm was used as the light source, and the “on” and “off” states of the UV illumination were controlled by a mechanical chopper. The flexible photodetectors fabricated on mica substrates (thickness of 25 µm) were fixed onto two motorized stages for acquiring signals at different bending angles, the cyclic bending–unbending tests were carried out using a caliper. All the above measurements were conducted in a dark room in ambient conditions.

## Conflict of Interest

The authors declare no conflict of interest.

## Supporting information

Supplementary

Supplementary
