# Supplementary material for: Single‐Step Selective Laser Writing of Flexible Photodetectors for Wearable Optoelectronics
Source: Adv Sci (Weinh). 2018 Jun 6;5(8):1800496. doi: 10.1002/advs.201800496 (PMC6097153; doi:10.1002/advs.201800496)
Supplement: Supplementary file 1 — Supplementary [file ADVS-5-1800496-s002.pdf]

Copyright WILEY-VCH Verlag GmbH & Co. KGaA, 69469 Weinheim, Germany, 2018.

ADVANCED SCIENCE
Open Access

Supporting Information

for *Adv. Sci.,* DOI: 10.1002/advs.201800496

Single-Step Selective Laser Writing of Flexible Photodetectors for Wearable Optoelectronics

Jianing An, Truong-Son Dinh Le, Chin Huat Joel Lim, Van Thai Tran, Zhaoyao Zhan, Yi Gao, Lianxi Zheng, Gengzhi Sun, and Young-Jin Kim**

Copyright WILEY-VCH Verlag GmbH & Co. KGaA, 69469 Weinheim, Germany, 2016.

Supporting Information

Single-step selective laser writing of flexible photodetectors for wearable optoelectronics

Jianing An, Truong-Son Dinh Le, Chin Huat Joel Lim, Van Thai Tran, Zhaoyao Zhan, Yi Gao, Lianxi Zheng, Gengzhi Sun, and Young-Jin Kim**

Dr. J. An, V. T. Tran, Dr. Z. Zhan, Y. Gao, Prof. Y.-J. Kim
School of Mechanical and Aerospace Engineering, Nanyang Technological University
50 Nanyang Avenue, 639798, Singapore
E-mail: yj.kim@ntu.edu.sg
T.-S. D. Le, C. H. J. Lim
Singapore Centre for 3D Printing, School of Mechanical and Aerospace Engineering, Nanyang Technological University
50 Nanyang Avenue, 639798, Singapore
Prof. L. Zheng
Department of Mechanical Engineering, Khalifa University of Science, Technology and Research
Abu Dhabi, 127788, United Arab Emirates
Prof. G. Sun
Key Laboratory of Flexible Electronics (KLOFE) & Institute of Advanced Materials (IAM), Jiangsu National Synergetic Innovation Center for Advanced Materials (SICAM), Nanjing Tech University (NanjingTech)
30 South Puzhu Road, Nanjing, 211816, P.R. China
E-mail: iamgzsun@njtech.edu.cn

1. Chemical reactions of the precursor film upon laser irradiation.

   Laser-induced reduction of GO to rGO and laser-induced thermal decomposition of zinc acetate dihydrate to zinc oxide.

$$\mathrm{GO} \xrightarrow{\text{photoreduction}} \mathrm{rGO} \tag{1}$$

$$\mathrm{Zn(CH_3COO))_2 \cdot 2H_2O} \overset{\Delta}{\rightarrow} \mathrm{Zn(OH)_2 + 2CH_3COOH} \tag{2}$$

$$\mathrm{Zn(OH)_2 \rightarrow ZnO + H_2O} \tag{3}$$

2. Construction of the smart photoswitch system.

   An instrumentational amplifier circuit was built with Op Amps IC (LM124N, STMicroelectronics) to amplify the signal of photodetector (Figure S6). Under UV illumination, the resistance of photodetector reduces and results in a voltage difference (V_{IN}) of the Wheatstone bridge. V_{IN} was amplified to V_{OUT} with the gain of this circuit being

$$\frac{V_{\mathrm{OUT}}}{V_{\mathrm{IN}}} = \left(1 + 2 \times \frac{R_5}{R_6}\right) \times \left(\frac{R_2}{R_1}\right) = \left(1 + 2 \times \frac{100\ \mathrm{k}}{10\ \mathrm{k}}\right) \times \left(\frac{100\ \mathrm{k}}{10\ \mathrm{k}}\right) = 210$$

   The amplified voltage V_{OUT} was used to drive the LED.

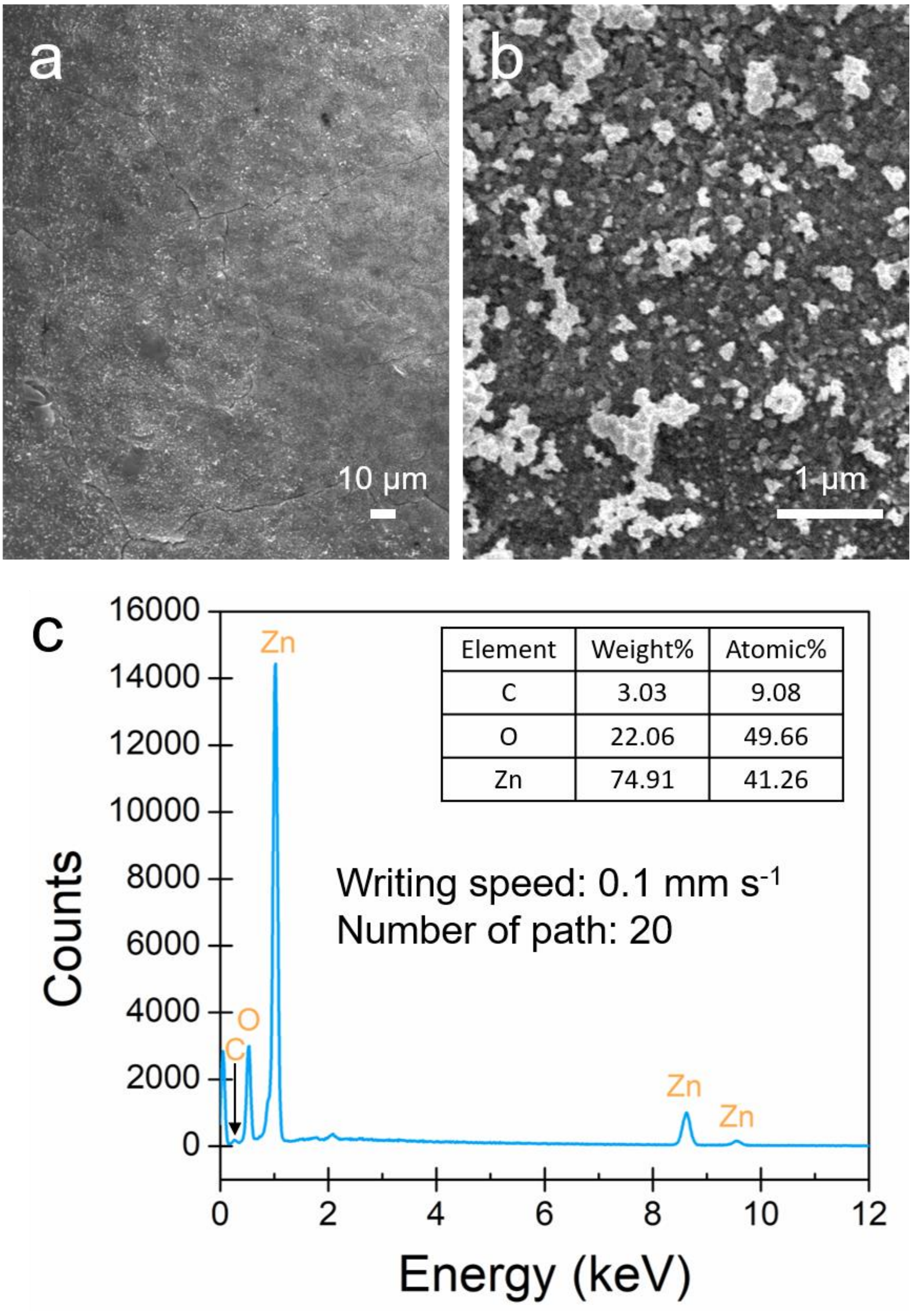

Figure S1. Carbon ablation when elongating the exposure time to laser irradiation. The film was written at the speed of 0.1 mm s^{-1} and repeated by 20 times. a) Low- and b) high-magnification SEM images showing the morphology of the as-written film. c) EDS spectrum of the as-written hybrid. The inset presents the element analysis in weight and atomic percentage.

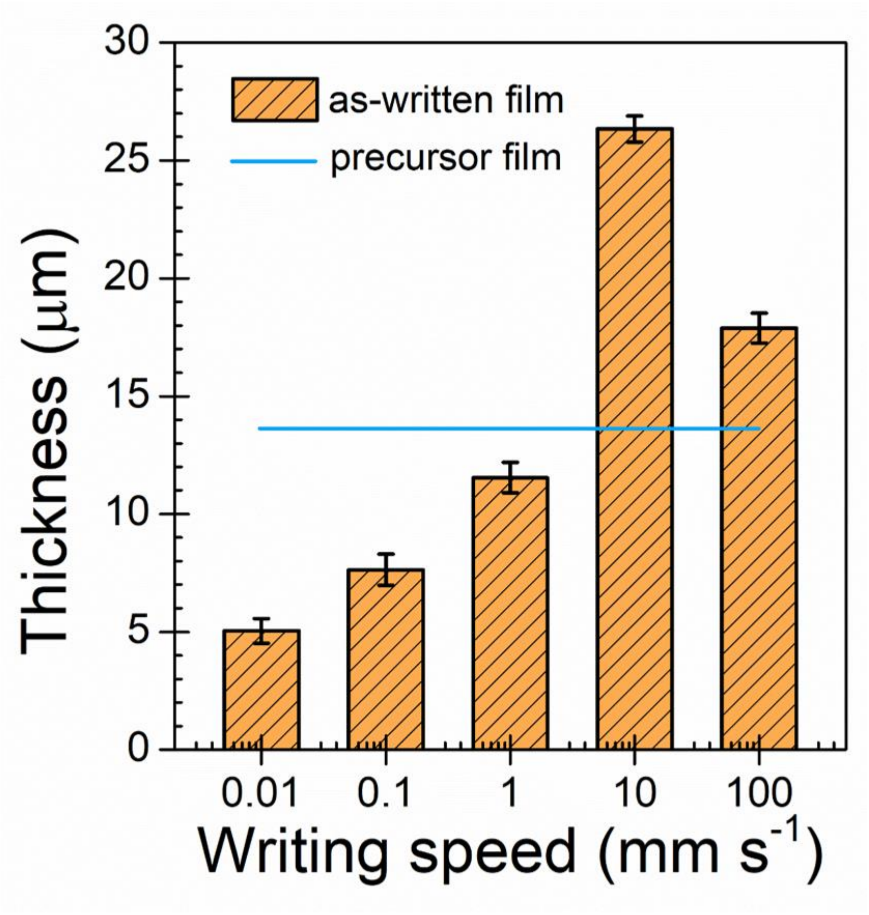

Figure S2. Plot of thickness change of the hybrid film written with varied speeds. The error bars result from re-sampling. The blue line indicates the thickness of the pristine precursor film.

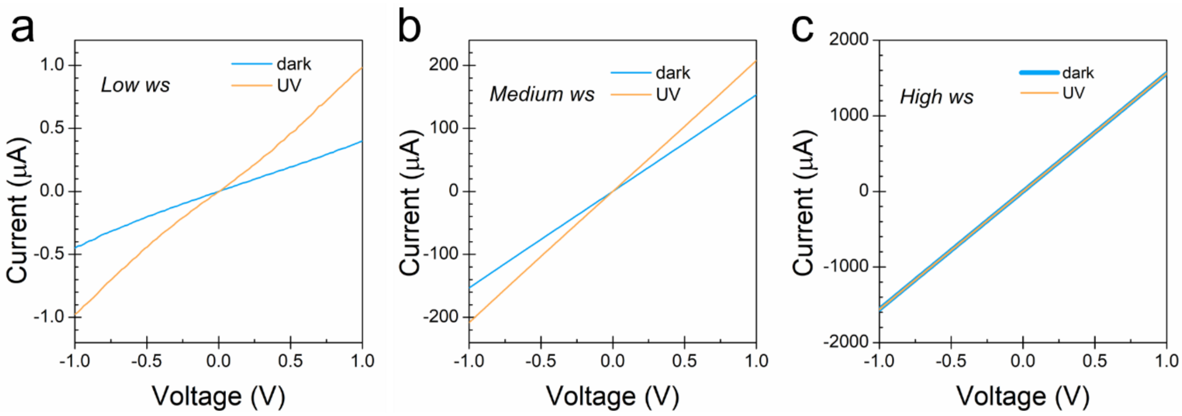

Figure S3. The *I-V* curves of the rGO-ZnO hybrids produced with a) low, b) medium, and c) high writing speeds in the dark and under UV illumination, respectively.

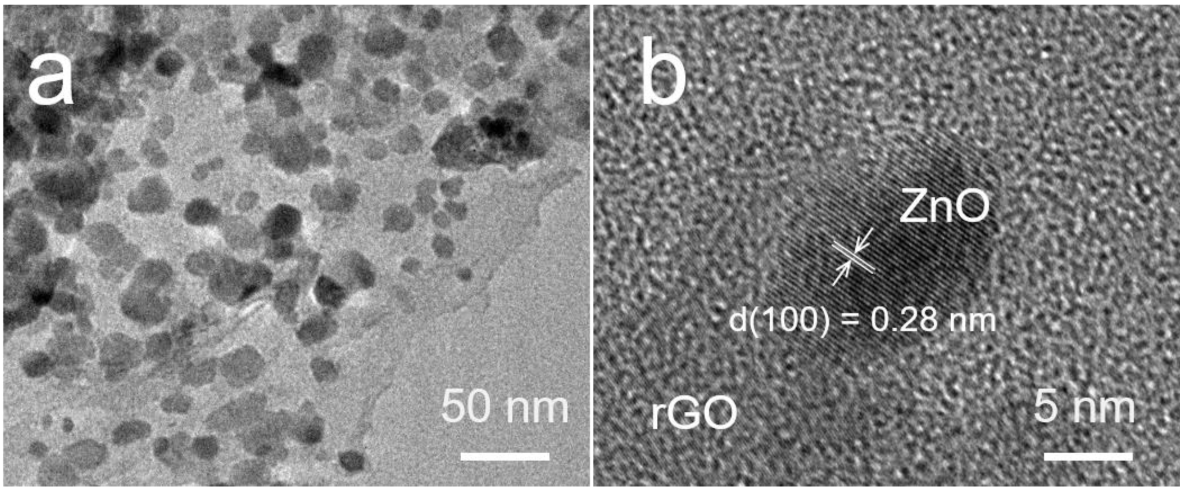

Figure S4. a) Transmission electron microscopy (TEM) image of rGO nanosheet anchored with ZnO nanoparticles. b) High resolution TEM image showing the rGO-ZnO interface.

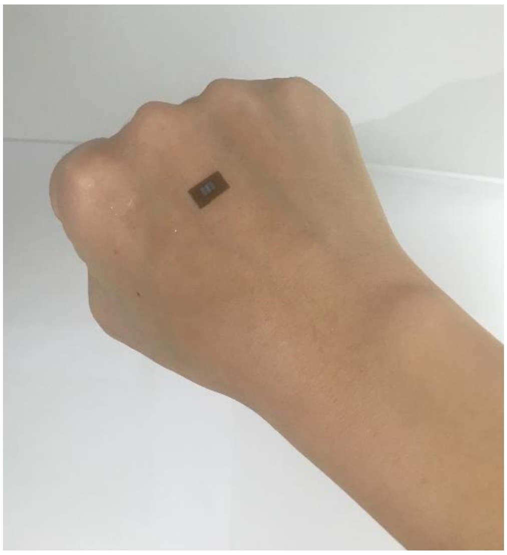

Figure S5. Photograph of a flexible photodetector conformally attached on a human hand.

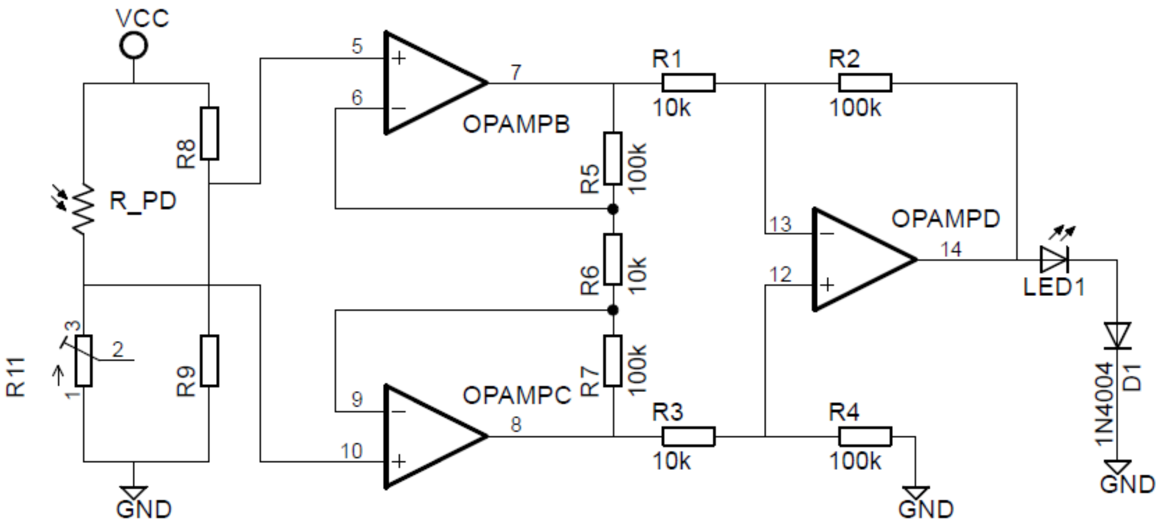

Figure S6. The configuration of the photoswitch system. R_{PD} denotes the resistance of the rGO-ZnO photodetector.

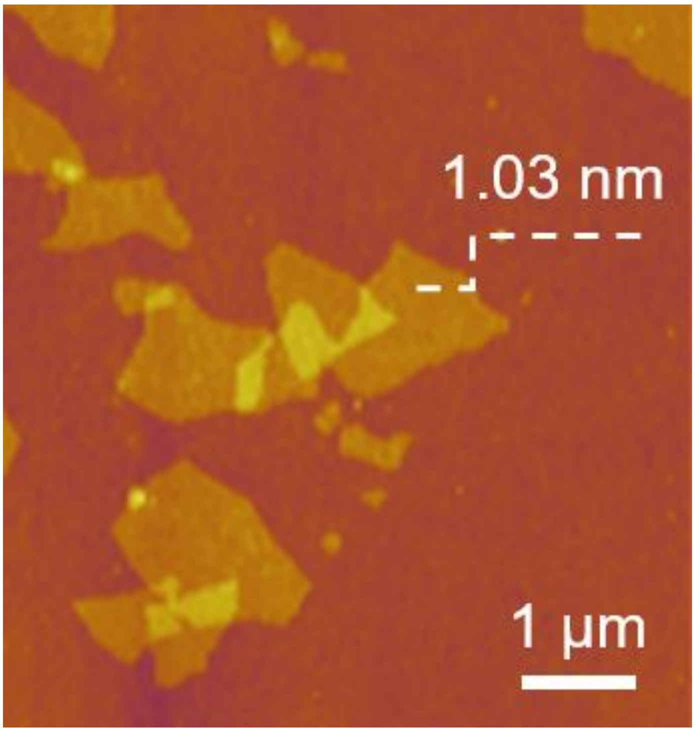

Figure S7. AFM image of the GO nanosheets.

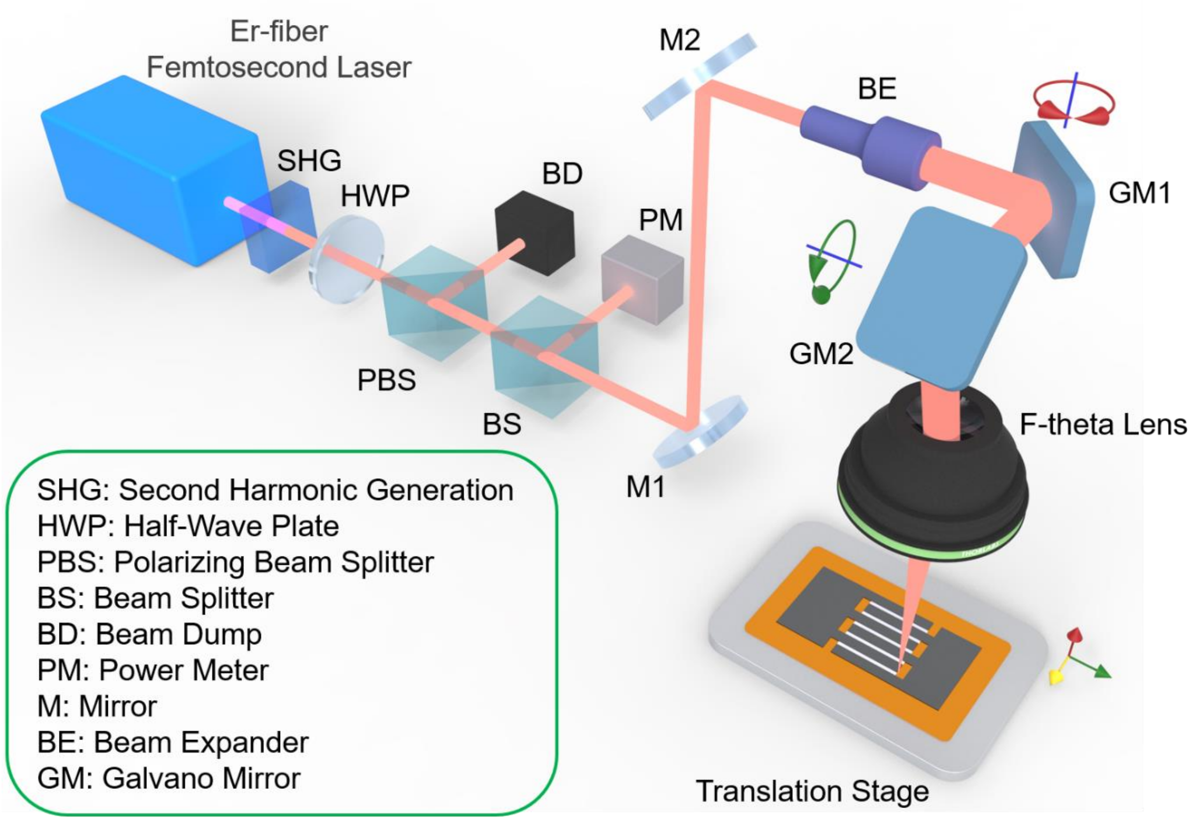

Figure. S8. Schematic illustration of the FsLDW setu
